# Cancer Treatment Closer to the Patient Reduces Travel Burden, Time Toxicity, and Improves Patient Satisfaction, Results of 546 Consecutive Patients in a Northern Italian District

**DOI:** 10.3390/medicina59122121

**Published:** 2023-12-04

**Authors:** Luigi Cavanna, Chiara Citterio, Patrizia Mordenti, Manuela Proietto, Costanza Bosi, Stefano Vecchia

**Affiliations:** 1 Casa di Cura Piacenza, Internal Medicine and Oncology, Via Morigi 3, 29121 Piacenza, Italy; 2 Department of Oncology and Hematology, AUSL Piacenza, Via Taverna 49, 29121 Piacenza, Italy; c.citterio@ausl.pc.it (C.C.); p.mordenti@ausl.pc.it (P.M.); m.proietto@ausl.pc.it (M.P.); c.bosi@ausl.pc.it (C.B.); 3 Pharmacy Unit, AUSL Piacenza, Via Taverna 49, 29121 Piacenza, Italy; s.vecchia@ausl.pc.it

**Keywords:** travel burden, cancer patients, time toxicity, rural patients

## Abstract

*Background and Objectives*: The distance to cancer facilities may cause disparities by creating barriers to oncologic diagnosis and treatment, and travel burden may cause time and financial toxicity. *Materials and Methods*: To relieve travel burden, a program to deliver oncologic treatment closer to the patient was initiated in the district of Piacenza (Northern Italy) several years ago. The oncologic activities are performed by oncologists and by nurses who travel from the oncologic ward of the city hospital to territorial centres to provide cancer patient management. This model is called Territorial Oncology Care (TOC): patients are managed near their home, in three territorial hospitals and in a health centre, named “Casa della Salute” (CDS). A retrospective study was performed and the records of patients with cancer managed in the TOC program were analysed. The primary endpoints were the km and time saved, the secondary endpoints: reduction of caregiver need for transport and patient satisfaction. *Results*: 546 cancer patients managed in the TOC program from 2 January 2021 to 30 June 2022 were included in this study. Primary endpoints: median km to reach the city hospital: 26 (range 11–79 km) median time: 44 min (range 32–116); median km to reach the territorial clinicians in the TOC program: 7 (range 1–35 km), median time: 16 minutes (range 6–54), *p* < 0.001. Secondary endpoints: 64.8% of patients who needed a caregiver for the city hospital could travel alone in the TOC program and 99.63% of patients were satisfied. *Conclusions*: The results of this retrospective study highlight the possibility of treating cancer patients near their residence, reducing travel burden and saving time.

## 1. Introduction

Despite significant advancements in diagnosis and treatment in cancer, disparities between cancer patients still persist [1,2]. Most of the research on the topic is focused on racial and socioeconomic disparities with little reported evidence about the effect of the burden of travel from a patient’s residence to health care providers [3,4]. Patients with cancer must overcome many challenges of a psychological, economic, social, and obviously of sanitary nature [5], and routine cancer clinic appointments can represent further difficulties that require time not only for visits and receiving direct cancer-related treatment, but also require additional time for travel, parking, and other transit needs [6]. Several studies have documented that the travel burden (measured as the travel distance or travel time) can result in delays in diagnosis and can negatively influence the choice of treatment for a variety of common cancers [7,8,9,10]. The most relevant objectives of health policies are to improve the quality, safety, patient satisfaction, and health care efficiency, as previously reported [5,11] A review, performed by our group, showed that travel burden negatively influences the stage at diagnosis, appropriate treatment, outcome, and quality of life in cancer patients [5]. In this review we found that in almost all of the analysed studies, patients who lived far from hospitals and had to travel more than 50 miles had a more advanced stage at diagnosis, lower adherence to encoded treatments, a worse prognosis, and a worse QoL. These four aspects are all very important for patients and for health care policies and costs. The cancer stage at presentation significantly influences treatment planning, as well as the short- and long-term prognosis. A diagnosis at an earlier stage can allow for less invasive, more efficacious, and less costly management. The travel time also can be considered a direct cost of cancer treatment that is usually borne solely by the patients and their families. As such, the time costs associated with travel are an important component of the full economic burden of cancer. Travel can be of particular importance for socioeconomically disadvantaged persons because the time costs associated with care can strain limited resources. Also, lower provider accessibility or transportation barriers can result in longer travel times for low-income individuals. In addition, the worse prognosis for patients living farther from treating hospitals could have been because the compliance with treatment or the follow-up program was suboptimal and transportation to the health care provider can be perceived as a barrier to care and can limit patients’ compliance with treatment [5]. With the objectives of reducing time and travel related burdens for cancer patients in the district of Piacenza (Northern Italy), the oncology and haematology department of the Azienda Sanitaria of Piacenza in agreement with the Health Authority, established a new territorial organization program for delivering oncologic management and treatment near the residence of cancer patients that has been operating since 2004. We named this program “territorial oncology care” (TOC). Reports related to patients with cancer treated near their residence have been previously reported [12,13]. In the present retrospective series of 546 cancer patients treated closer to their home, we examined the time and km of travel spared, cancer patient satisfaction, and the need to be accompanied by a caregiver. This study aimed to describe the feasibility of cancer care delivery closer to the patients.

## 2. Materials and Methods

Our territorial-based model of care for oncological (TOC) patients includes outpatient clinics, located closer to the patients home, in three community hospitals; this model of care has been implemented since July 2016; in fact, similar clinical activities have been established in a health centre named “Casa della Salute” (CDS) in an area without community hospitals, thus allowing this oncological service to be provided throughout the entire Province of Piacenza. The oncological outpatient management in the province of Piacenza is allocated in dedicated areas within the three community hospitals and the CDS.

Oncological activities are performed by oncologists and by specialized nurses who travel from the Hospital of Piacenza to the peripheral hospitals and to the CDS. This model provides the same modality of care of those of the referring hospital for cancer patients since they are managed by the same specialists (oncologists and specialized nurses).

In this model, the patient with cancer is managed by the outpatient service of one of the nearest community hospitals or the CDS; the oncologists take care of all treatments: the drugs are scheduled with a specific computer program, according to the same computerized model in place at the oncology referral department, Hospital of Piacenza. All anticancer therapies are prepared at the antiblastic drug unit of the referring hospital, Unità Farmaci Antiblastici (UFA), where all the treatment schedules are registered and recorded so that the traceability of the administered therapies is ensured. The dedicated area for oncologic management in the community hospital and in the CDS is similar to an outpatient clinic where medical services are offered by oncology nurses and by oncologists.

The oncologic activities performed in the community hospitals and in the CDS are:

− Patient’s clinical evaluation, non-invasive diagnostic tests (blood tests, electrocardiogram, ultrasound (US) of chest-abdomen and soft tissues);
− Diagnostic-therapeutic us-guided procedures (fine needle aspiration-biopsy, paracentesis, thoracentesis).

All cases treated at the CDS and in the community hospitals are collegially discussed with the oncological medical and nursing staff of the oncology department, of the referring Hospital of Piacenza in order to share therapeutic choices and to increase the possibility of offering experimental clinical trials to these patients. For diagnostic techniques and surgical treatment not available in peripheral hospitals, cancer patients are sent to the central referring hospital after diagnosis and are evaluated by the multidisciplinary oncology group; after surgical treatment, they can return to receiving medical treatment near their residence. The referring physicians inform the patients that can chose the site of medical treatment: closer to their residence or at the referring city hospital. In addition, each patient was free to choose whether to continue his/her treatment in the TOC program or to remain at the city hospital.

The primary endpoints of this study were the km saved with treatment closer to patient’s home and the average travel times gained with this organization, evaluated by calculating the distance from the residence of the patients to the outpatient setting closer to their home, compared with the distance to reach the oncology unit of the referring hospital, using Google Maps. The secondary endpoints were (1) the reduction of need for caregivers to drive patients to reach the nearest outpatient setting, compared to the need for caregivers to reach the central hospital of Piacenza, (2) the rate of satisfaction with treatment nearest to the residence of patients analysed with a dedicated questionnaire that used a 5 item Likert-type scale ranging from “not satisfied at all/completely disagree” to “very satisfied/strongly agree” [14]. This questionnaire was administered after two months of treatment and at the end of the planned therapy.

For the present study, we analysed all of the files containing the schedules of patients managed in the community hospitals and in the CDS; all files have been electronically recorded at the UFA of the Local Health Authority of Piacenza, and contain the clinical data of cancer patients such as sex, age, type of cancer, stage, type, and line of treatments. For this research, we focused on intravenous, intramuscular, and target therapies delivered close to patients’ homes.

The patients’ personal data, residence, primary disease site, histology, disease stage, and detailed information about the administered therapy, i.e., administration route (intravenous, intramuscular), line of treatment (neoadjuvant, adjuvant, treatment for metastatic disease), and type of anticancer drugs (chemotherapy, immunotherapy, hormones, ...) have been collected in these files and analysed.

Here we report the data of oncologic patients treated closer to their home from 2 January 2021 to 30 June 2022.

### Statistical Analysis

Quantitative variables were described by median and interquartile range (IQR) and qualitative variables were described by absolute and percentage frequencies. Normality was checked for all continuous variables. Comparisons of covariates were conducted using the Mann–Whitney test for the continuous variables (distance and time). All analyses were performed using RStudio version 3.6.0 statistical software (Boston, MA, USA) with two-sided significance tests and a 5 % significance level.

## 3. Results

A total of 552 patients with cancer were asked to participate the TOC program, of these, 546 patients (98.91%) on active treatment were managed in the TOC program from 2 January 2021 to 30 June 2022 and were included in this study, 6 patients (1.09%) preferred to be treated at the referring city hospital, so the “uptake” of participation to this program was very high. The clinical and demographic characteristics of these 546 patients are reported in Table 1.

The median age was 70.5 IQR (63–77.25) years, range 30-89 years. There were 277 (50.73%) women and 269 (49.27) men. The types of cancers were gastroenteric (35.71%), breast (23.26%), genitourinary (15.39%), and non-small cell lung cancer (13.55%). The majority of patients showed metastatic disease (69.96%), while 30.04% of patients showed early stage disease, 164 patients (30.04%) were treated in a neoadjuvant or adjuvant setting, and 303 (55.49%) in the first line for metastatic disease; 422 patients (77.29%) were treated with chemotherapy and 44 (8.06%) with immunotherapy, 7 (1.28%) with chemo and immunotherapy, and 73 (13.37%) with target therapy (Table 1). The total visits of patients to the community hospitals and CDS were 3,371 and the total infusional therapies were 5676 from 2 January 2021 to 30 June 2022 (Table 2).

Primary endpoints: the median distance from the patient’s residence to the oncology unit of the referring city hospital (Piacenza) was 26 (IQR 23–38) km (range 11–79 km) while it was 7 (IQR 3–12) km (range 1–35 km) to the community hospital and to the CDS (*p* < 0.001). The median time for the travel to the oncology unit and parking at the referring hospital of Piacenza was 44 (IQR 39–52) min (range 32–116) while it took 16 (16–23) min (range 6-54) to reach the community hospitals and the CDS (*p* < 0.001) (Table 3).

**Figure 1:**
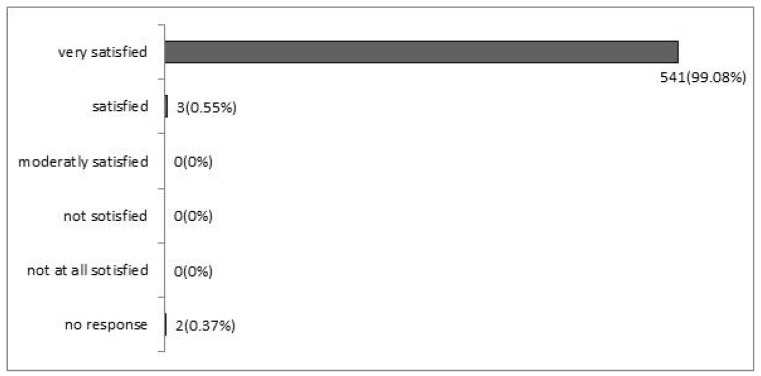
Response to the question “how satisfied are you with the oncologic management closer to your home?”.

Secondary endpoint: 99.63% of patients were satisfied or very satisfied to receive oncological treatment near their residence (Figure 1) and an average of 65% (64.8%) of patients who needed a caregiver to reach the oncology unit of the referring city hospital could travel alone to the territorial outpatient centre closer to the patients. Only two patients (0.37%) chose to continue treatment at the city hospital after 4 and 6 months, respectively, for family reasons.

## 4. Discussion

We are aware that in the 21st century, oncologists can deliver personalized medicine using the molecular profile of patients’ cancer genomes to optimize disease management [15]. The position paper by the European Society of Medical Oncology (ESMO) reports that at the centre is the patient, with personalized medicine offering the promise of delivering safe and efficacious targeted cancer treatment [15]. However, patients with cancer must overcome many economic, social, psychological, and even family barriers to obtain the needed diagnosis and treatment [5]. Increasingly, cancer studies have identified distance to care as a very important measure of access to visits and oncologic treatments that impact health outcomes [1,7,8,9,10]. A workforce analysis by the American Society of Clinical Oncology (ASCO) showed that only 3% of medical oncologists practice in rural areas whereas 20% of the US population resides in rural areas and over 70% of counties in the United States do not have medical oncologists [2,16]. In Europe geographic disparities in access to cancer care have been reported in France [17,18,19], Scotland [20], Sweden [21,22], and Italy [23,24]. A study from Switzerland showed that medical oncologists take treatment schedules into consideration that can diminish difficulties in access to care [25]. However, the reasons for these preferences for treatment chosen to reduce the burden of travel and save time do not necessary fit with evidence-based medicine, and the impact of this choice on cancer treatment outcomes should be considered. It must be emphasized that with our program, the cancer patients treated closer their residence received the same anticancer treatment schedules as at the referring oncology ward of the city hospital. In addition, this program, in our oncology network, provided the opportunity for patients treated closer to their home to participate clinical trials with innovative cancer drugs. It is well known that participation in randomized controlled trials is associated with improved cancer survival; however, many trials require frequent examinations. Thus, the travel burden can exclude patients from trials owing to the distance of their residence from the trial centre. In our region, with the provincial oncological network, rural oncologic patients have ready access to oncological services and can be treated under the supervision of a medical oncologist who travels to the peripheral units. This network hosts a centralized unit for the preparation of anticancer drugs with a computerized system that facilitates participation in randomized clinical trials. The burden of travel from the patient’s residence to the care provider may result in psychosocial and financial distress and may represent an important issue that can influence the quality of life and outcome of cancer patients [5]. In this contest, oncology care should aim to improve the quality of life of cancer patients, providing patients and their caregivers with the possibility of realizing their full potential, whether the cancer is curable or not, and oncologists cannot ignore the problems related to the travel burden of cancer patients. It is known that a cancer diagnosis presents a particularly significant problem for rural residents and providers, depending on the stage and site, cancer typically requires expensive evaluations from multiple specialists, and the disease and treatment can make it difficult for patients to drive or even work. It must be emphasized that rural populations are more socioeconomically disadvantaged and less educated than their urban counterparts [26]. Regional disparities in health have required attention in many countries, disparities in resource allocation can affect access to care and can increase patient movement to hospitals outside of their area of residence for care [27]. For these reasons, travelling may increase expenses arising out of repeated tests and treatments along with other costs that can arise due to various travel expenses and lost opportunities [28]. 

Since cancer studies have increasingly identified distance to care as an important measure of access to care with impact in health outcomes [7,8,9,10], and in a review of the literature, it was highlighted that in almost all of the examined studies, patients who lived far from hospitals had a more advanced stage at diagnosis, lower adherence to encoded treatments, and, above all, a worse quality of life and a worse prognosis [5]. Wardley et al. [29] introduced the concept of “flexible care” to describe treatment performed outside of the oncology ward, for example in a primary care setting, community care centres, or any other applicable setting, with the objective of reducing the travel burden, giving time back to the patient, and avoiding time toxicity. We are aware that geographic disparities in access to cancer care can also play a significant role in access to screening methods as reported by Simion L. et al. [30]. In fact, as reported by the authors, a Romanian woman is diagnosed with cervical cancer every two hours, and Romania has the highest rural population among European Union member states [30]. We previously reported that for 54 patients residing in rural areas treated between July 2016 and July 2017, the average distance to the closest outpatient centre was 21 km versus 82 km to the referring hospital of Piacenza. This shorter distance allowed for a much shorter average travel time for treatment (16 min to the territorial centre versus a 93 min round trip to the referring hospital [12]. We remember that time at home has become an important outcome for many patients, not just cancer patients [5,6,31,32]. For cancer patients, time at home was considered important, above all, for patients with limited life expectancies or/and limited mobility. However, we agree with Banerjee R et al. [6] that time at home should also be considered an important objective for managing cancer patients during the earlier phases of the disease and its treatment. Treatment changes introduced during the COVID-19 pandemic, such as the use of telehealth in oncology [32], modified cancer therapy infusion frequencies [33,34], and oral treatment options when feasible, have allowed cancer patients and their caregivers to spend more time at home [35]. Yet a greater number of patients with metastatic cancer is being treated actively with anticancer and supportive drugs for a prolonged period [36,37,38,39]. Many of these patients are elderly, with additional comorbidities such as hypertension, chronic obstructive pulmonary disease, coronary disease, diabetes and others [40]. They need continuous primary care as well as oncology care. A study of patients with lung cancer explored the influence of travel burden (measured as travel distance and travel time) on clinical outcomes and significant differences were discovered in the overall survival of patients with lung cancer depending on the travel distance and travel time to the treating oncological facility. The median overall survival was significantly lower in patients with a higher travel burden, despite having similar clinical and pathological characteristics [3]. Our study demonstrated that the treatment of patients with cancer in outpatient territorial centres located closer to their home can significantly reduce travel distance and the time needed to reach the referring ward for treatment. In addition, a very high rate of satisfaction with treatment in the territorial centre was registered, and most patients who needed caregivers to reach the referring oncology ward of the city hospital were able to travel independently to reach the territorial centre. There is a shift today towards care being provided closer to the patient’s residence, which has the following benefits: reducing travel time and the associated costs and providing a sense of freedom for both patients and caregivers [41].

Our study demonstrated that the treatment of cancer patients in outpatient territorial centres located closer to their home can significantly reduce the travel distance and time needed to reach the referring ward for treatment, since the median distance to the outpatient centre was 7 (range 1–35) km versus 26 (range 11–79) km to the referring ward of the city hospital. The shorter distance resulted in much shorter average travel times for treatment, 16 (range 6–54) min to the territorial centre versus 44 (range 32–116) min to travel to the referring hospital.

However, our study has some limitations: its retrospective in nature, it was conducted in only one territorial district of Northern Italy, this territorial model can count on pre-existing peripheral hospitals, and the medical and nurse staff was very motivated to provide oncologic care closer to the home of the patients.

In conclusion we believe that the results of our retrospective study highlight the possibility of treating cancer patients in territorial structures near their residence, with advantages for the patients themselves, their caregivers, and the entire community.

## 5. Conclusions

Travel distance to cancer facilities may cause disparities by creating barriers to oncologic diagnosis and treatment and may cause time toxicity; however, the medical community has neglected this issue. To relieve travel burden and improve time and cost savings for cancer patients and caregivers and to allow for patients to receive adequate oncologic care, an innovative program to provide oncologic treatment near patients’ residences in the district of Piacenza (Northern Italy) was initiated several years ago.

During an 18 month period, a total of 546 patients with cancer on active treatment were managed in this program. The median distance from the patients’ residence to the oncology unit of the hospital of Piacenza was 26 km (range 11–79 km), while it was 7 km (range 1–35 km) to the territorial centre (*p* < 0.001). The average travel time was 44 min (range 32–116) to reach the city hospital and 16 min (range 6–54) to reach the territorial centre (*p* < 0.001). In addition, 99.63% of patients were satisfied, and 64.8% of patients who needed a caregiver to reach the city hospital could travel alone to reach the territorial centre.

We believe that the results of our retrospective study highlight the possibility of treating cancer patients in territorial structures near their residence; it has advantages for patients, their caregivers, and the entire community.

## Figures and Tables

**Table 1:** Clinical and demographic characteristics of the 546 cancer patients treated closer to their home.

Characteristics	Patients (*n* = 546)
Sex *n* (%)	
female	277 (50.73)
male	269 (49.27)
Median age (IQR) (range)	70.5 (3–77.25) (30–89)
Primarytumor location *n* (%)	
gastroenteric	195 (35.71)
genito-urinary	84 (15.39)
breast	127 (23.26)
lung	81 (14.83)
head and neck	20 (3.66)
other	39 (7.14)
Setting of treatment *n* (%)	
early/localized/neoadjuvant/adjuvant	164 (30.04)
metastatic line	382 (69.96)
1’line	303 (79.32)
subsequent lines	79 (20.68)
Therapy *n* (%)	
intravenous chemotherapy	422 (77.29)
intravenous immuno/chemiotherapy	7 (1.28)
intravenous immunotherapy	34 (6.22)
intramuscolar immunotherapy	10 (1.83)
target therapy	73 (13.37)

**Table 2:** Number of visits to the territorial outpatient structure and number of infusional anticancer therapies in 546 consecutive cancer patients treated closer to their home from 2 January 2021 to 30 June 2022.

*n*. visits from 2 January 2021 to 31 December 2021	2254
*n*. infusion therapies from 2 January 2021 to 31 December 2021	3884
*n*. visits from 2 January 2022 to 30 June 2022	1117
*n*. infusion therapies from 2 January 2022 to 30 June 2022	1792
total visits	3371
total infusion therapies	5676

**Table 3:** Median distance (kms) and time (minutes) used to travel from the residence to the nearest Community Hospital/CDS or to the referral city Hospital of Piacenza. (CDS: Casa della Salute, IQR: interquartile range).

	Distance from Patient’s Residence to Center of Care		*p*-Value	Time		*p*-Value
	Median (IQR) (kms)	Range (kms)		Median (IQR) (min)	Range (min)	
Residence—nearest Community Hospital/CDS	7 (3–12)	1–35	<0.001	16 (16–23)	6–54	<0.001
Residence—referral city hospital	26 (23–38)	11–79		44 (39–52)	32–116	

## Data Availability

The data that support the findings of this study are available from the corresponding author, [L.C.], upon reasonable request.
